# Cost effectiveness of a novel swallowing and respiratory sensation assessment and a modelled intervention to reduce acute exacerbations of COPD

**DOI:** 10.1186/s12890-025-03615-y

**Published:** 2025-04-09

**Authors:** Isabella Epiu, Christine R. Jenkins, Norma B. Bulamu, Andreas Kuznik

**Affiliations:** 1 Prince of Wales clinical School, The University of New South Wales, Sydney, NSW 2052 Australia; 2 The George Institute for Global Health, University of New South Wales, Sydney, NSW Australia; 3 Department of Thoracic Medicine, Concord General Hospital, Sydney, NSW Australia; 4 Concord Clinical School, University of Sydney, Sydney, NSW Australia; 5 College of Medicine and Public Health Flinders Health and Medical Research Institute, Flinders University, Adelaide, SA Australia; 6 Health Economics and Outcomes Research, Regeneron Pharmaceuticals, Tarrytown, NY USA; 7 Kabale University School of Medicine, Kabale, Uganda

**Keywords:** Chronic respiratory diseases, Dysphagia, Cost effectiveness analysis, Airway sensation, Swallowing, SwaRSA

## Abstract

Swallowing impairment observed in ~ 20% of people with Chronic Obstructive Pulmonary Disease (COPD) may increase the risk of aspiration pneumonia and acute exacerbations. We designed a decision analytic model to assess the cost-effectiveness of the Swallowing and Respiratory Sensation Assessment (SwaRSA) tests and swallowing rehabilitation to reduce COPD exacerbations. We believe that swallowing rehabilitation to improve coordination of swallowing and breathing may reduce exacerbations in people with COPD.

From the Australia health system perspective, we assessed the cost effectiveness of four tests relative to standard of care, or no testing, over a time horizon of one year. The SwaRSA tests assessed relative to a standard of care arm of no testing: included the Eating Assessment Tool (EAT-10) score, Swallowing Capacity of Liquids, Tongue Strength Assessment, and Respiratory Sensation Assessment, in people with moderate to severe COPD. Outcome measures were COPD exacerbations per year, which were converted into quality adjusted life years (QALYs). Model inputs including costs, test sensitivities and specificities, COPD exacerbation risks, and exacerbation-related utilities were derived from published sources. Our assumptions on the costs, recovery, and risk reduction are based on the available data on pulmonary rehabilitation in COPD.

Relative to no-SwaRSA, three individual testing strategies were found to be cost-effective at incremental cost effectiveness ratio per QALY ranging from $27,000 to $37,000 AUD assuming a willingness to pay of $50,000 AUD. The EAT-10 and the tongue strength were the two dominant options on the cost-effectiveness frontier. Model results were robust to variations in one-way and probabilistic sensitivity analyses.

In COPD, SwaRSA modelling suggests that self-assessment with the EAT-10 and subsequent intervention is highly cost-effective relative to no-SwaRSA.

**Supplementary Information:**

The online version contains supplementary material available at 10.1186/s12890-025-03615-y.

## Introduction

Patients with moderate-to-very severe Chronic Obstructive Pulmonary Disease (COPD) represent a considerable economic burden despite the current efficacious treatments available [1]. COPD is among the most common non-communicable diseases, affecting 329 million people with a global economic cost of US$ 2·1 trillion [2]. Annual COPD costs in the USA are expected to increase to 40 billion USD per year [3]. Australia spent about AUD$ 831.6 million in 2020-21 on management of COPD; accounting up to 18% of disease expenditure on respiratory conditions, and 0.6% of total disease expenditure [4]. Over half of this cost was for hospital care [4]. We believe that a significant portion of this cost could be avoided by reducing exacerbations.

Studies have documented swallowing difficulties (dysphagia) in 15–20% of COPD patients [5], and in one study, 56% of hospitalized patients with a primary diagnosis of COPD were diagnosed with oropharyngeal swallow disorder [6]. In this study of patients with acute exacerbations of COPD (AECOPD), 17% of COPD patients with exacerbations aspirated [6]. During video-fluoroscopy in another study, 25% of people with COPD aspirated, and faced a greater number of hospitalizations and deaths over 36 months [7, 8].

Dysphagia is not only common in COPD [9–12], but it is a significant economic burden, associated with up to 60% higher economic costs than people with normal swallowing, and increased length of stay in hospitals [13, 14]. It is estimated that 235 million older adults will suffer from a swallowing disorder, or dysphagia worldwide by 2050 [15]. Disrupted swallowing function is observed in 15% of people over 65 years old, and is often reported as dysphagia [16]. Oropharyngeal dysphagia can result in penetration of particles into the larynx, aspiration into the trachea, or presence of oropharyngeal residue [17].

Impaired sensation and perception of respiratory loads is linked to poor outcomes of respiratory diseases [18–21]. Our investigations of people with COPD found impaired sensation of inspiratory loads [22, 23], and tongue strengths that were lower than the published weighted averages; here, around 30% of the COPD participants showed clinical signs of aspiration [12]. Additionally, COPD patients have disrupted swallowing-breathing coordination in everyday life [9], and this could predispose them to aspirate, further increasing the exacerbation rate. Such aspirations can lead to aspiration pneumonia, worsening symptoms, or AECOPD, and this group with a high risk of aspiration could benefit from close monitoring and swallowing training.

We therefore designed a decision analytic model to assess the cost effectiveness of implementing inexpensive swallowing and respiratory sensation assessments (SwaRSA) to identify high-risk COPD participants. The tests included in this evaluation were part of a larger study to assess neuro-motor impairments in people with COPD and healthy ageing [24, 25]. Before, no regular assessment of swallowing difficulties existed for people with COPD. However, rapid swallowing assessment in stroke or other suspicious cases of aspiration commonly require high tech instrumental swallowing evaluations with expensive equipment like video-fluroscopy or fibreoptic endoscopic evaluation of swallowing, and the presence of a qualified specialized speech therapist [26, 27].

Our SwaRSA decision analytic model includes bed-side tests and a modelled intervention to prevent AECOPD. We believe that early identification of high-risk patients and swallowing co-ordination training may reduce exacerbations in people with COPD.

## Methods

We designed a decision analytic model to assess the cost-effectiveness of the SwaRSA tool. We modelled the effect of SwaRSA (swallowing and respiratory sensation assessment and a rehabilitation intervention) to aid in the diagnosis of dysphagia, an independent predictor of COPD exacerbation risk. Since SwaRSA is not part of routine clinical care, the strategy was compared against a strategy of no SwaRSA in the control arm of the model. In our modelling, the SwaRSA arm received an intervention while the no SwaRSA did not.

The model time horizon was limited to 12 months of follow-up, therefore making it most suitable for a relatively simple decision tree rather than a long-term Markov extrapolation, because the underlying clinical data that served as the basis to inform the dysphagia related exacerbation risk was similarly limited to 12 months of follow-up [38]. The model outcomes included in the end nodes of the decision tree included the total expected cost of exacerbation related treatment, exacerbation-dependent quality of life estimates, as well as the cost of SwaRSA related screening and clinical intervention only in the intervention arm of the model. Our model-based economic evaluation and reporting complies with the Consolidated Health Economics Reporting Standards (CHEERS) guidelines [28].

### Choice of model

The decision analytic model was used in this cost-effectiveness analysis as a robust tool to guide policy makers on this essential swallowing and respiratory sensation assessment and dysphagia training in COPD in the absence of a costed clinical trial. The one-year time horizon is a crucial component in decision analytic modelling for COPD, especially for evaluating the immediate effects of interventions. It also accounts for seasonal variations in COPD exacerbations, ensuring relevant and actionable insights for decision-makers. By addressing uncertainties through sensitivity analyses, these models provide robust evidence for short-term intervention planning [29–32].

Decision analytic modelling is a systematic approach used in health economics, offering a structured methodology to evaluate healthcare interventions for COPD, especially when empirical data is limited. Give the chronic nature of COPD, such models synthesize data from diverse sources such as clinical trials, observational studies, and expert opinions to simulate decision-making processes, assess costs, benefits, and risks, and estimate long-term clinical and economic outcomes. Frameworks like Decision trees, Markov models, and Microsimulations are frequently employed to calculate metrics such as incremental cost-effectiveness ratios (ICERs). Sensitivity analyses further bolster the reliability of decision-making by exploring the robustness of outcomes under varying assumptions. This approach ensures healthcare decision-makers have robust, quantitative evidence to guide resource where other data are limited [33–38].

Decision trees are particularly effective for short-term healthcare interventions with discrete outcomes. They provide graphical representations of decision-making processes, allowing for the calculation of expected values by incorporating probabilities, costs, and health outcomes, such as quality-adjusted life years (QALYs), further enhancing the flexibility of decision analytic modelling [32, 39]. Over decades, such decision analytic models have evolved into indispensable tools, enabling decision-makers to extrapolate short-term clinical data into long-term outcomes, ensure economic efficiency, and enhance the delivery of healthcare services [38, 40, 41].

### Target population and subgroups

Our target population for this decision analytic model were patients with moderate-to-very severe COPD who represent a considerable economic burden despite the current efficacious treatments available from frequent hospitalization many related to exacerbations of the disease.

### Study setting and perspective

This is cost-effectiveness evaluation was part of a larger study to assess neuro respiratory impairments in people with COPD [12, 22, 23, 25]. In people with moderate to severe COPD, we assessed swallowing dynamics, inspiratory muscle responses to sudden airway occlusion, and neural perception including airway sensation, and respiratory related evoked potentials from EEG studies [12, 22, 23, 25]. Our Australian subjects had lower tongue strength compared to published weighted averages, ~ 30% of the COPD group showed clinical signs of airway invasion, and a delayed perception of loads [12, 22, 23, 25].

### Comparators

Here we modelled the use of SwaRSA and a hypothetical rehabilitation intervention to reduce COPD exacerbations compared to the standard of care. The current standard of care does not involve any routine swallowing or respiratory sensation assessment. In the model, this was referred to as no-SwaRSA.

The SwaRSA model included four tests:- the questionnaire-based eating assessment tool (EAT-10) score, swallowing capacity of liquids from the timed water swallow test (TWST), tongue strength assessment from the Iowa Oral Performance Instrument (IOPI), and respiratory sensation assessment in a group of patients with moderate to severe COPD.

We assumed that a hypothetical rehabilitation to improve swallowing dynamics [42], if implemented, early could help reduce AECOPD.

### Time horizon

The decision analytic modelling employed a time horizon of one year as the model considered exacerbations in COPD over 12 months. This time horizon was most suitable for a relatively simple decision tree rather than a long-term Markov extrapolation, because the underlying clinical data that served as the basis to inform the dysphagia related exacerbation risk was similarly limited to 12 months of follow-up.

### Discount rate

There was no discounting considered due to the one year timeframe employed in this model.

### Choice of health outcomes

The outcome of interest was AECOPD in the subjects over the one-year period. The exacerbations markedly increase costs of care through admissions, medications, and oxygen support. Therefore, the purpose of this model was to reduce such costs associated with AECOPD and improve the quality of life of people with COPD.

### Model structure

**Fig. 1:**
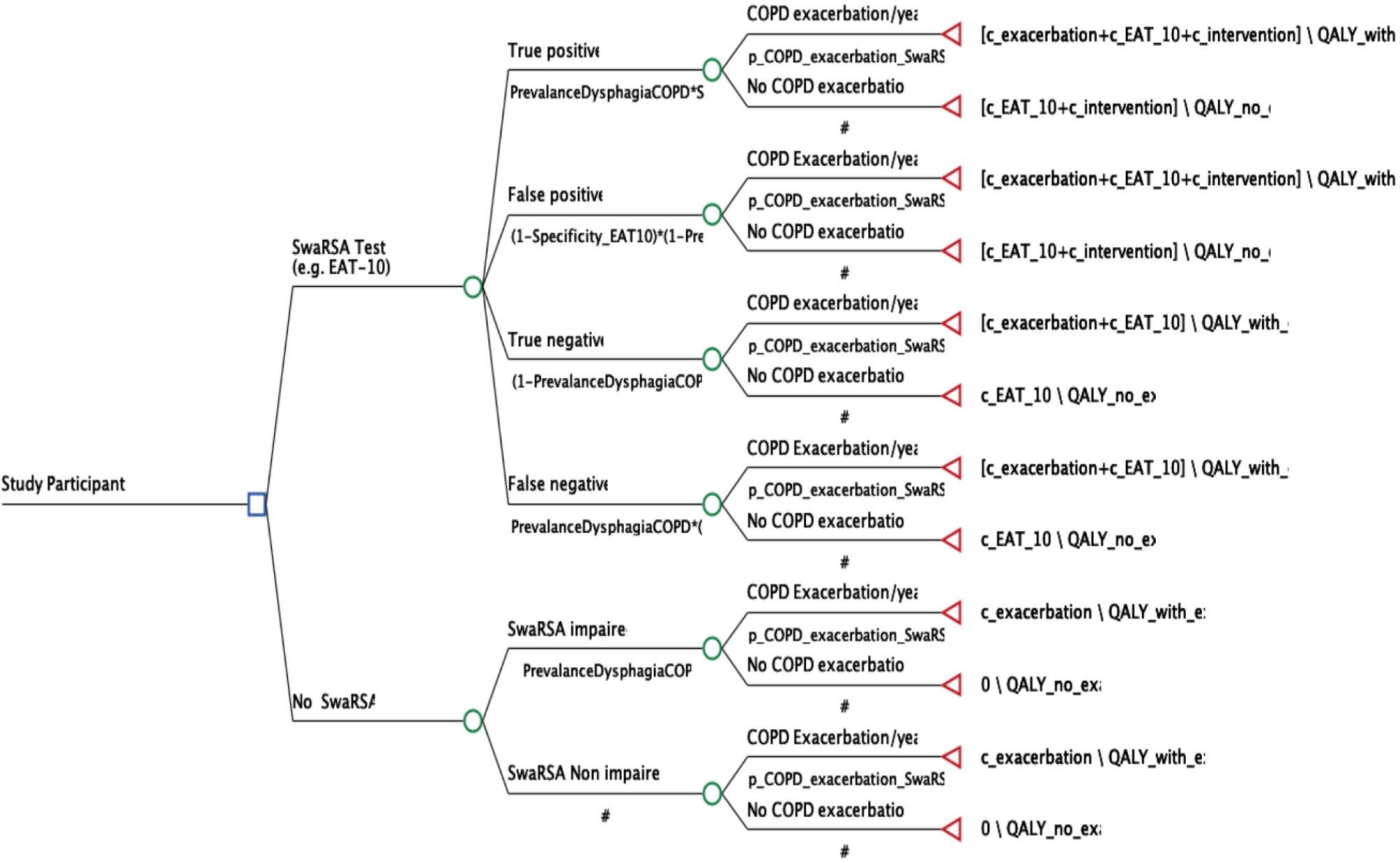
Showing cost effectiveness SwaRSA model decision tree. Abbreviations: EAT-10– Eating Assessment Tool, Exa– exacerbations, C– Costs, P– Probability, QALY = Quality adjusted life years, SwaRSA = Swallowing and Respiratory Sensation Assessment. Equations: True positives (prevalence X sensitivity), False positive ( [1-Specificity] X [1-prevalence]), True negative ([1-Prevalence] X Specificity), and False Negative (Prevalence X [1-Sensitivity])

The analysis was conducted from the Australian payor perspective. All costs included in the model are in Australian dollars (AUD). This SwaRSA model calculates the sum of all costs, and quality adjusted life years (QALYs) in COPD with or without exacerbation, at a willingness to pay threshold of $50,000 per QALY in 2021 Australian dollars. We used a 1-year horizon in the model. Health states in the model included COPD exacerbation versus no-exacerbation (see Fig. 1. for a structure of the model).

Using the accuracy of each test (Table 1.), and the prevalence estimates of COPD exacerbations and dysphagia, we estimated the true positives (prevalence X sensitivity), false positive ([1-prevalence] X [1-specificity]), true negative ([1-prevalence] X specificity), and false negative (prevalence X [1-sensitivity]). Figure 1. Shows inputs for one test, EAT-10, however, a similar model was used for all the other tests. In this decision tree model, study participants were grouped into SwaRSA test or no-SwaRSA test arms where the SwaRSA test arm also received a modelled intervention and the no SwaRSA did not. All model inputs are described above and in Table 1.

**Table 1:** Model inputs sensitivity and specificity of SwaRSA tests

Model Input	Sensitivity	Specificity	Probability	Reference
EAT-10	0.85	0.82		[43]
IOPI	0.92	0.39		[44]
TWST	0.86	0.50		[45]
Respiratory Sensation	0.80	0.15		[46]
Probability of exacerbation with dysphagia	-	-	0.87	[54]
Probability of exacerbation no dysphagia	-	-	0.39	[54]

Abbreviations: EAT-10–Eating Assessment Tool, IOPI: Iowa Oral Performance Instrument, TWST-Timed Water Swallow Tes

### Measurement of effectiveness

The tests used in this *SwaRSA* model were demonstrated to be clinically effective in these published studies, and results for sensitivity and specificity are listed in Table 1 [43–46]. Additional effectiveness measures used in this model were QALYs with a utility at baseline in people living with COPD estimated at 0.65 QALYs (ranging from 0.50 to 0.80), and 0.22 QALYs (range 0.00 to 0.50) for a respiratory exacerbation [47–50].

Patients with exacerbations were assigned a QALY of 0.526 calculated by the QALY at baseline plus QALY during exacerbations using the Eq. (0.65*(37/52) + 0.22*(15/52)) with an average of three exacerbations per year and 5 weeks recovery period. From Donaldson et al. study, people with COPD experienced up to 3 exacerbations per year [51]. Further reports revealed that 75% of COPD patients returned to baseline peak respiratory flows after 35 days following exacerbation [52].

In the model control group, we assumed a 20% prevalence of dysphagia, with the remaining 80% being dysphagia free. The probability of exactly three exacerbations is 87% among COPD patients with dysphagia, with the remaining 13% assumed to experience zero exacerbations. The corresponding probability for patients without dysphagia is 39%. Hence, the weighted average expected number of exacerbations in the control group is 0.2*((0.87*3)+(0.13*0)) + 0.8*((0.39*3)+(0.61*0) = 0.522 + 0.936 = 1.458. In the intervention group, the risk of exacerbation is reduced by 46% among the 20% of patients with dysphagia, hence: 0.54*0.2*((0.87*3)+(0.13*0)) + 0.8*((0.39*3)+(0.61*0) = 0.282 + 0.936 = 1.218.

### Currency, price date and conversion

The current AUD exchange rates of February - March 2022 were used.

### Model inputs

The costs of implementing the EAT-10 questionnaire were estimated at $11.72, tongue strength using the Iowa Oral Performance Instrument (IOPI) at $37.02, TWST for swallowing liquids at $23.69, and load perception test at $73.16 per experiment (See Supplementary Table 1).

The SwaRSA model input costs, sensitivities and specificities were from published sources; see Table 1. The accuracy of EAT-10 was set at 85.0%, and 82.0%, tongue strength 91.6% and 38.5%, TWST 85.5% and 50.0%, for sensitivity and specificity respectively [43–45]. However, for the load perception test of respiratory sensation in COPD, the sensitivity and specificity (80.0% and 15.0%) were derived by comparing the load perception results among participants that recorded clinical signs of aspiration during the swallowing tests [46, 53]. Additional model inputs, according to Cvejic et al. [54], the probability of exacerbation with dysphagia was set at 0.87 and exacerbation without dysphagia at 0.39 (see Table 1).

### Assumptions

From Donaldson et al. study, people with COPD experienced up to 3 exacerbations per year [51]. We used the higher end of exacerbation frequency because the “frequent exacerbators” often have multiple hospital admissions and a rapid decline in quality of life [55], and this constitutes a major cost to the health care system.

For the SwaRSA model, we assumed that the exacerbation risk can be decreased in patients that test positive by means of the appropriate swallowing rehabilitation and training intervention. The estimated risk reduction on the acute COPD exacerbations after the swallowing intervention was set at 46%. This is consistent with a clinical trial conducted in Australia, in which there was a 46% reduction in admissions due to AECOPD following pulmonary rehabilitation [56].

### Model analysis

Patients that were positively identified by the SwaRSA test were assigned an intervention in the model with a cost and treatment effect to reduce the risk of future exacerbations. The cost of this intervention was derived from a Canadian study that listed this cost as CAD$ 1,092, which we converted to AUD$ 1,208 at current exchange rates [57]. The total cost per exacerbation has been reported at £3,726 in a UK study [58], which we similarly converted to AUD$ 7,040 using current exchange rates. In the comparator arm, we populated probabilities and costs to provide a baseline of the COPD outcomes without any SwaRSA tests and no intervention, Fig. 1.

### Analytic methods & model outputs

The SwaRSA model calculated the sum of all costs, sum of all QALYs, cost per QALY gained and we derived a cost-effectiveness frontier with all tests. The incremental costs, incremental effectiveness, and ICERs were also calculated following a hypothetical swallowing training and rehabilitation in the model. We also performed a one-way sensitivity analysis, and a probabilistic sensitivity analysis (PSA) with 10,000 iterations following several variations for the EAT-10 test (See Table 2).

**Table 2:** Model output showing cost effectiveness of SwaRSA

Dominance	Strategy	Cost AUD$	Incr Cost, AUD$	Eff QALY	Incr Eff: QALY	ICER
undominated	EAT-10	3334	-87.9	0.598	0.0084	-10,419
abs. dominated	No -SwaRSA	3421		0.590		
ext. dominated	TWST mls/s	3653	231.7	0.598	0.0085	27,293
undominated	Tongue Strength -IOPI	3758	336.5	0.599	0.0091	37,000
abs. dominated	Respiratory Sensation Assessment	4059	637.1	0.598	0.0079	80,213

Abbreviations: EAT-10– Eating Assessment Tool, IOPI: Iowa Oral Performance Instrument, COPD– Chronic Obstructive Pulmonary Disease; QALY = Quality adjusted life years, SwaRSA = Swallowing and Respiratory Sensation Assessment; TWST– Timed Water Swallow Test

## Results

**Fig. 2:**
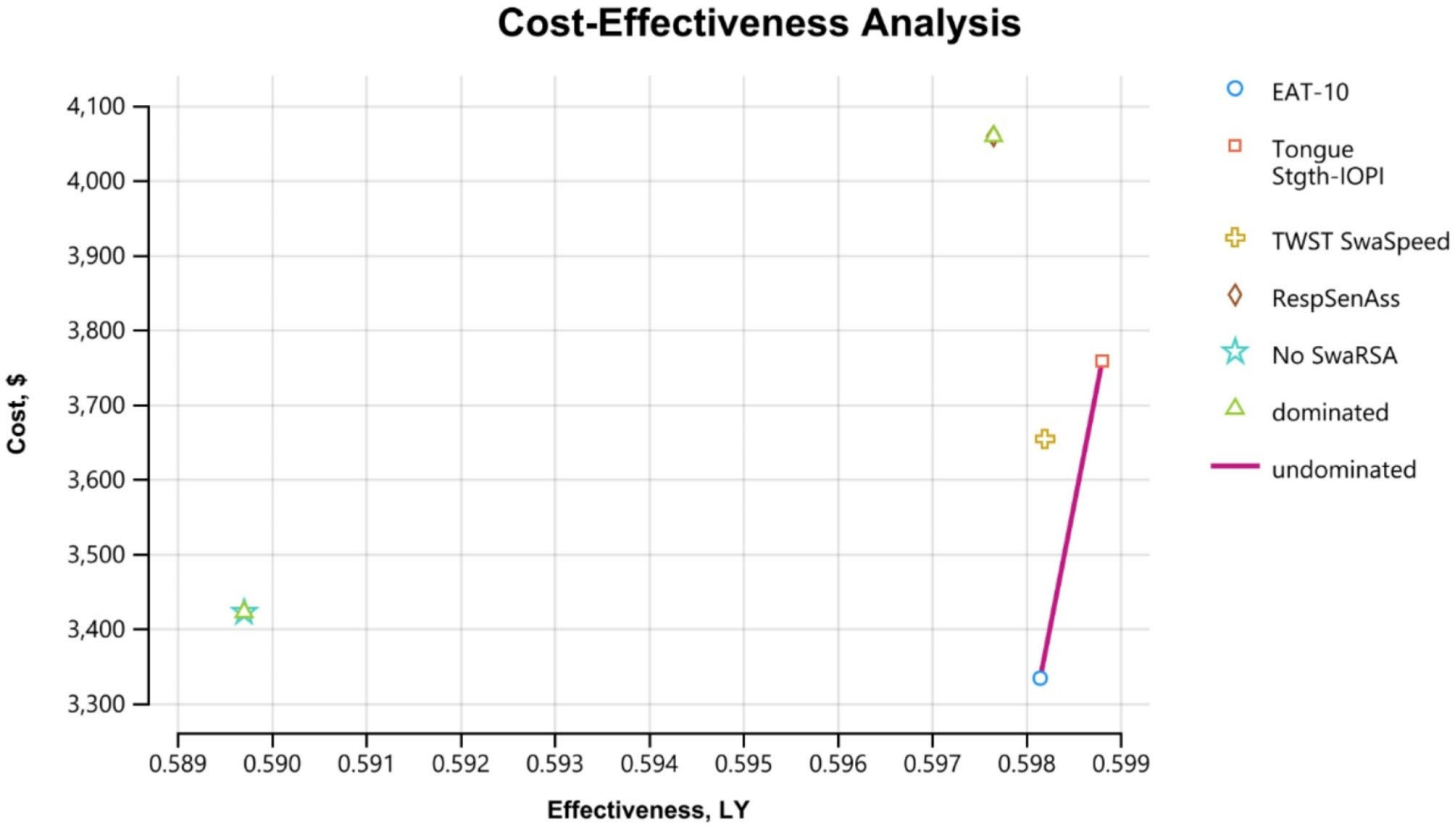
Showing cost effectiveness analysis of SwaRSA. Abbreviations: EAT-10– Eating Assessment Tool, IOPI: Iowa Oral Performance Instrument, Stgth– Strength; COPD– Chronic Obstructive Pulmonary Disease; QALY = Quality adjusted life years, SwaRSA = Swallowing and Respiratory Sensation Assessment; TWST– Timed Water Swallow Test; ResSenAss - Respiratory Sensation Assessment

The modelled intervention following the SwaRSA assessment were more effective than no-SwaRSA in reducing exacerbations and improving quality of life in COPD and increased QALYs by 0.01 per annum, see Fig. 2. With no-SwaRSA, there were 0.59 QALYs, however this increased to 0.60 with the EAT-10, TWST and the respiratory sensation assessment and a hypothetical swallowing rehabilitation in this decision analytic model, Table 3. The individual QALY outcomes for each test was 0.598, 0.598, 0.599, 0.598 for EAT-10, TWST, Tongue Strength, and Respiratory Sensation Assessment respectively, versus 0.590 for no-SwaRSA, Table 3.

**Table 3:** Model variations for the sensitivity analysis– EAT-10 vs. no-SwaRSA

ITEM	LOWER LIMIT	UPPER LIMIT
Cost EAT-10	5.86	17.58
Sensitivity EAT-10	0.75	0.95
Prob COPD Exacerbation_SwaRSA_Impaired	0.77	0.97
Specificity EAT-10	0.72	0.92
Prevalence Dysphagia COPD	0.3	0.1
Cost intervention	604	1812
QALY_NO_Exacerbation	0.55	0.75
Cost_exacerbation	3520	10,660
Risk Reduction_intervention	0.25	0.75
QALY_With_exacerbation	0.43	0.63

Abbreviations: EAT-10– Eating Assessment Tool, IOPI: Iowa Oral Performance Instrument, COPD– Chronic Obstructive Pulmonary Disease; QALY = Quality adjusted life years, SwaRSA = Swallowing and Respiratory Sensation Assessment

Compared to no-SwaRSA, three of the four included strategies, i.e., TWST swallowing capacity, tongue strength IOPI, and Respiratory Sensation Assessment, were estimated to have positive incremental costs of ranging from AUD$230 to AUD$630. The one exception was the lowest cost strategy, the EAT-10. Factoring in the subsequent exacerbation-related costs, the EAT-10 strategy showed an incremental cost of -$88 when compared to no-SwaRSA in this model.

This implies that the EAT-10 strategy was also the most cost-effective in the analysis set. On the cost-effectiveness plane (Fig. 2), the EAT-10 dominated no-SwaRSA, TWST swallowing capacity, as well as Respiratory Sensation Assessment. The only undominated strategy of IOPI tongue strength was associated with an ICER of AUD$647,000 relative to EAT-10, which far exceeds the assumed willingness to pay of AUD$50,000 per QALY in Australia. TWST swallowing capacity strategy was externally dominated with an ICER of AUD$6.4 million compared to the EAT-10 strategy. Notably, however, when compared to current standard of care of no-SwaRSA, TWST and tongue strength strategies were also cost-effective with ICERs of AUD$27,000 and AUD$37,000 respectively. The respiratory sensation assessment was not cost effective relative to no-SwaRSA with an ICER of $80,000, Table 3.

**Fig. 3:**
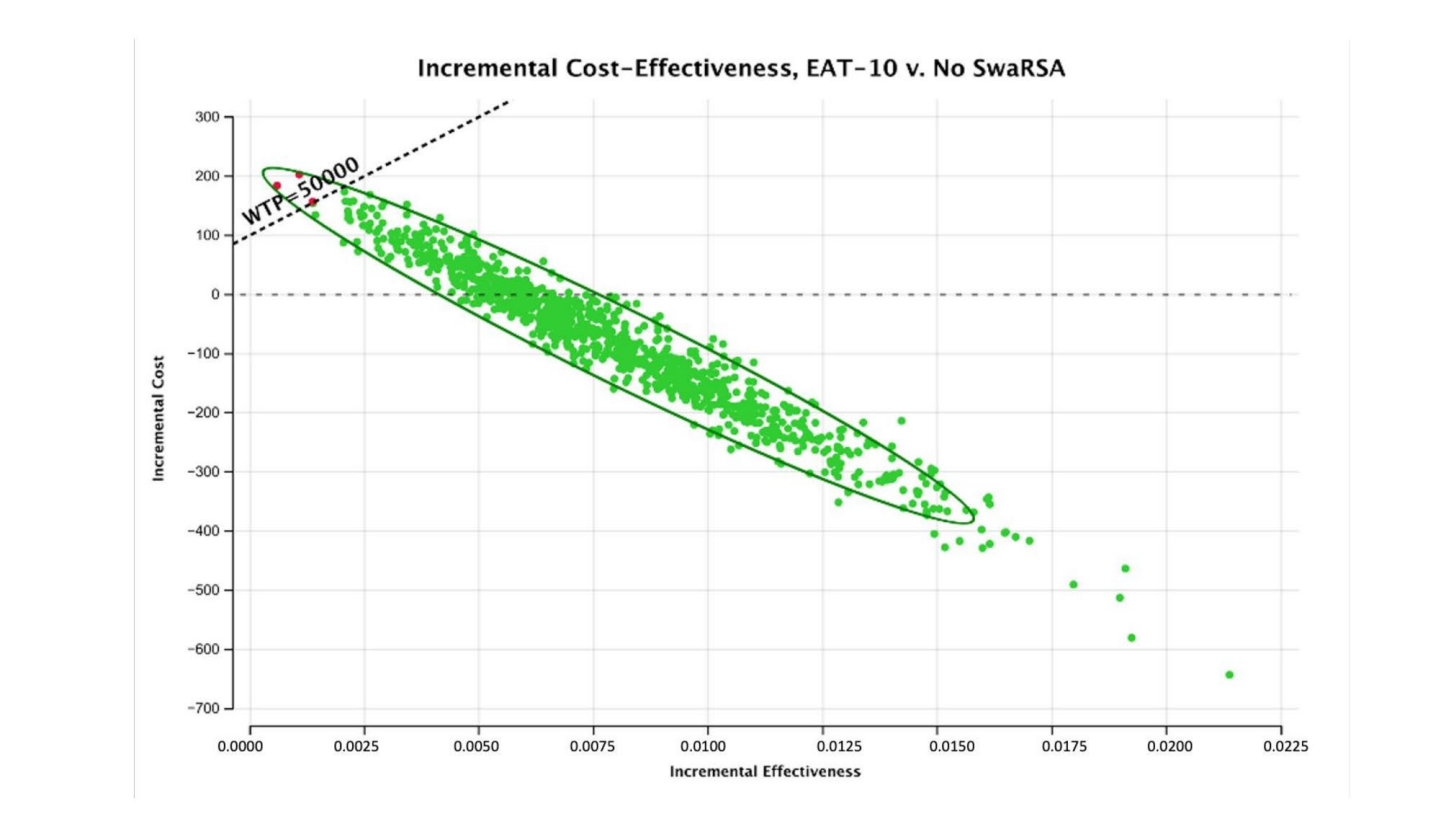
Incremental Cost-effectiveness of EAT-10 at a willingness to pay of 50,000$. Abbreviations: EAT-10– Eating Assessment Tool, Exa– exacerbations, C– Costs, P– Probability, SwaRSA = Swallowing and Respiratory Sensation Assessment

**Fig. 4:**
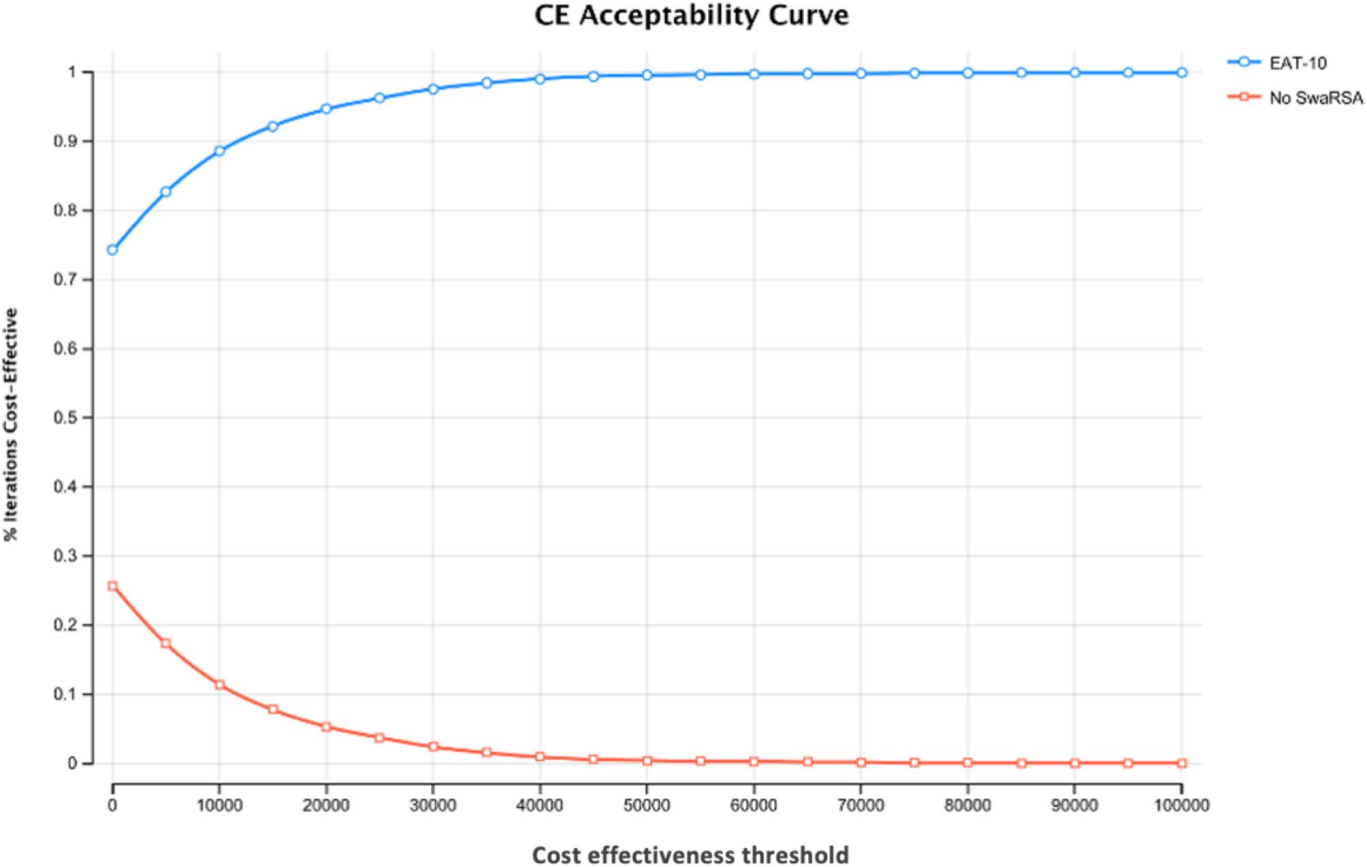
Acceptability curve. Abbreviations: EAT-10– Eating Assessment Tool, Exa– exacerbations, C– Costs, P– Probability, SwaRSA = Swallowing and Respiratory Sensation Assessment, CE– Cost effectiveness

Results from the probabilistic sensitivity analysis suggests that there is a high likelihood that the EAT-10 score is cost effective relative to no-SwaRSA as 99.6% of ICER iterations fell below the AUD$50,000 willingness to pay threshold (Fig. 3). Furthermore, the probabilistic results in Fig. 4 indicate that the favourable cost-effectiveness assessment is independent of the willingness to pay, as well over 70% of iterations yield an acceptable ICER for EAT-10 even at a willingness to pay of zero.

**Fig. 5:**
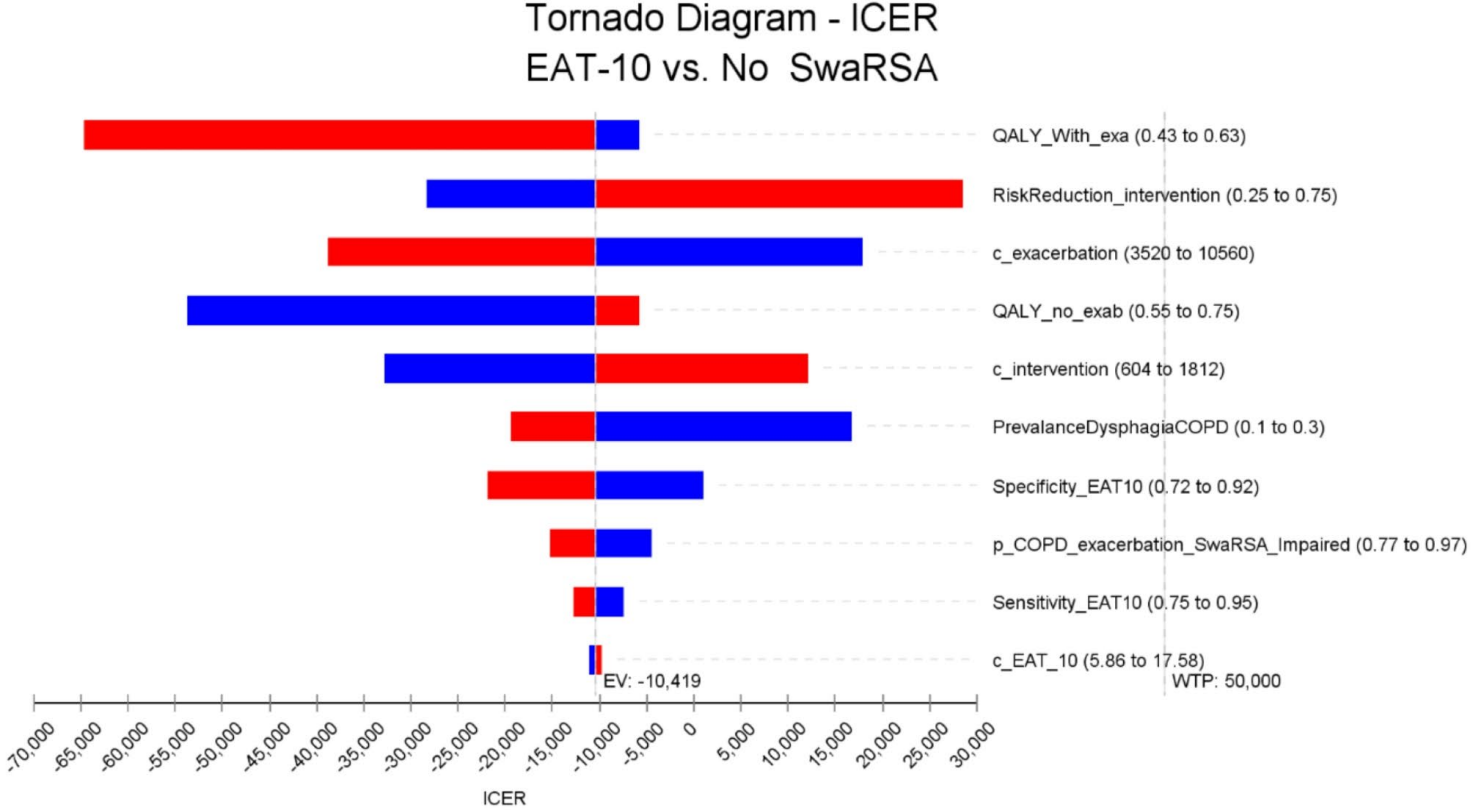
One-way sensitivity analysis. The width of the bars represents the variation of the model ICER outputs resulting from a change in the parameter values of a single input. The midpoint of the tornado diagram represents the base case ICER estimate. Abbreviations: EAT-10– Eating Assessment Tool, Exa– exacerbations, C– Costs, ICER- Incremental Cost effectiveness Ratio, P– Probability, QALY = Quality adjusted life years, WTP - Willingness to pay threshold, Exa– exacerbations, C– Costs, P– Probability, QALY = Quality adjusted life years, SwaRSA = Swallowing and Respiratory Sensation Assessment

The model was also robust to single variations in key model parameters. In one-way sensitivity analysis, we replaced one single base case model input with its lower and upper bound, while holding all other model parameters constant. Figure 5 displays the 10 inputs that the model was most sensitive to, ranked from largest to smallest deviation from the base case ICER for the assessment of EAT-10 versus no-SwaRSA. None of the 10 high value ICERs resulting from the more conservative model inputs exceeded the willingness to pay of $50,000 per QALY.

## Discussion

This is the first study showing the cost effectiveness of swallowing and airway sensation assessments with a modelled intervention in patients with COPD. We built a decision analytic model to illustrate the potential value of implementing the SwaRSA tests and an intervention to address AECOPD by comparing incremental costs and outcomes.

From this SwaRSA model all, the four tests followed by the training intervention were more effective than no-SwaRSA in reducing AECOPD however, EAT-10 scoring was the most cost-effective option in this decision analytic model. Over 99% of 10,000 PSA simulations resulted in an ICER less than $50,000 per QALY gained for EAT-10 and the modelled intervention versus no-SwaRSA with no swallowing intervention thus confirming the results.

From this model output, SwaRSA testing alongside an intervention appears accurate, relatively inexpensive, and effective in reducing the AECOPD. The EAT-10 strategy was both more effective and had a lower cost when compared to no-SwaRSA, making it the dominant strategy in this context.

In this model, we assessed four SwaRSA tests used in our neuro-respiratory trials including swallowing of liquid, tongue strength assessment, and airway sensation, rather than assessing swallowing of liquids alone [12, 25, 59]. To assess respiratory sensation, the load perception test using plugs was included in the decision tree [46, 53]. We determined the specificity and sensitivity of the load perception test by comparing the data in those who had clinical signs of aspiration during the TWST test. The load perception test showed a low specificity of 15% [46], thus its implementation in combination with other Swallowing Assessment tests could be examined to increase the combined sensitivity and specificity for early diagnosis of dysphagia and aspiration risk not only in COPD but across all high-risk groups.

There is an association between isometric orolingual pressure and swallowing pressure, but limited data exists [60, 61]. However, COPD commonly occurs in advanced ages, where people may also have muscle weakness [5, 62]. From our study [22], both the COPD groups and the healthy elderly age matched control groups had lower tongue strengths compared to published weighted averages, and this was associated with clinical signs of aspiration in about 30% of participants with COPD. From this SwaRSA model, swallowing training following tongue strength assessment with the IOPI was cost-effective. Infarct, a recent study shows that Interferential current transcutaneous electrical sensory stimulation (IFC-TESS) which stimulates the larynx and pharynx, improved sensory function in COPD [42], providing a targeted intervention that could improve swallowing in patients with COPD who are easily fatigued and struggle to perform swallowing exercises.

Aspiration in COPD may occur due to changes in breathing rate, reduced coordination of breathing with swallows, or dysfunctional upper airway–protective mechanisms [63, 64]. COPD patients with tachypnea may have more frequent ill-timed swallows that further increases their risk of aspiration. They usually inspire during, or immediately after swallowing, which increases their risk of aspiration [11, 65]. Swallowing with an altered rhythm of breathing is a risk factor for COPD exacerbations and this dysphagia affects disease progression [11, 62, 66]. However, from this SwaRSA model, implementation of cost-effective methods to identify, and then provide swallowing training for people at risk of aspiration is important particularly to improve breathing-swallowing coordination and reduce complications such as AECOPD.

### Limitations

The 46% estimated risk reduction [40] could be subject to regression to the mean. Also. the number of exacerbations in the control arm is reflective of the patients that were included in study [35] and may not be generalizable to all COPD populations.

This cost-effectiveness analysis is limited by a time factor and lack of funding, so we used a hypothetical model rather than a costed clinical trial. However, as in Fig. 3, modelling of the EAT-10 test costs were below the willingness to pay level, and its implementation in the SwaRSA model remained highly cost effective even after doubling the costs involved with 99% acceptability at a willingness to pay threshold of $50,000, see Figs. 3 and 4.

The underlying annual rates of AECOPD could range from 0.8 to 3.1 events per year [67], with varying recovery periods per participant. Participant adverse events, and exacerbations, could be documented, and used in a future cost-effectiveness analysis to test the results of this SwaRSA model in addressing AECOPD. Our assumptions on the costs, recovery, and risk reduction are based on the available data of pulmonary rehabilitation in COPD, and not swallowing training in people with COPD, which was lacking. Because the current SwaRSA model inputs were informed largely by published papers, a costed prospective randomised clinical trial is recommended. This modelling exposes the need for further research into targeted swallowing training for people with COPD in order to reduce AECOPD.

Beyond cost-effectiveness, decision analytic models integrate health-related quality of life measures, enabling the evaluation of healthcare interventions for COPD from a holistic perspective. By incorporating tools such as QALYs, these models facilitate the assessment of interventions’ value beyond clinical endpoints. Advanced techniques, such as probabilistic sensitivity analyses and cost-effectiveness acceptability curves used in this SwaRSA model, improve transparency and policy relevance, addressing uncertainties and guiding evidence-based healthcare decisions. This SwaRSA model could be adopted for decision analytic modelling of additional strategies beyond the four tests used in this model to address AECOPD.

## Conclusion

This study provides economic insight for policy makers and clinicians to include SwaRSA tests as regular screening in respiratory clinics and to provide swallowing retraining programs to reduce AECOPD. This novel approach to screening for swallowing impairment in COPD could be implemented as a regular test by testing for self-reported dysphagia scores using the EAT-10 followed by an appropriate swallowing training. In the absence of a costed clinical trial, this SwaRSA decision analytic model is a robust economic tool to guide implementation of EAT-10 scoring as a regular assessment for patients with COPD.

## Electronic supplementary material

Below is the link to the electronic supplementary material.

Supplementary Material 1

Supplementary Material 2

## Data Availability

Any additional data or the complete decision analytic model are available from the corresponding author on reasonable request.

## Operational Definitions

SwaRSA: Swallowing and Respiratory Sensation Assessment plus swallowing intervention
No-SwaRSA: No test and no intervention
Strategy: Refers to the test plus the swallowing intervention
