# Supplementary material for: Cost effectiveness of a novel swallowing and respiratory sensation assessment and a modelled intervention to reduce acute exacerbations of COPD
Source: BMC Pulm Med. 2025 Apr 9;25:165. doi: 10.1186/s12890-025-03615-y (PMC11980303; doi:10.1186/s12890-025-03615-y)
Supplement: Supplementary file 1 — Supplementary Material 1 [file 12890_2025_3615_MOESM1_ESM.docx]

Cost inputs for the SwaRSA tests

| ITEM SwaRSA | Details | Time (hours) | Unit Cost (AUD) | Cost per experiment (AUD) | Useful Lifetime /unit size |
| --- | --- | --- | --- | --- | --- |
|  |  |  |  |  |  |
| **1. EAT-10** |  |  |  |  |  |
| Technician/Research Assistant | 1.0 | 0.25 | 46.9 | 11.72 |  |
| TOTAL |  |  |  | 11.72 |  |
|  |  |  |  |  |  |
| **2. IOPI** |  |  |  |  |  |
| Technician/  Research Assistant | 1.0 | 0.5 | 46.9 | 23.44 |  |
| IOPI Machine | 1.0 |  | 3,050.0 | 0.59 | 10 years |
| IOPI Bulbs | 1.0 |  | 13.0 | 13.00 | Single use |
| TOTAL |  |  |  | 37.02 |  |
|  |  |  |  |  |  |
| **3. TWST** |  |  |  |  |  |
| Technician/Research Assistant | 1.0 | 0.5 | 46.9 | 23.44 |  |
| Cups (disposable) | 2.0 |  | 0.1 | 0.24 | Single use |
| Timer | 1.0 |  | 13.0 | 0.01 | 3 years |
| TOTAL |  |  |  | 23.69 |  |
|  |  |  |  |  |  |
| **4. Load perception** |  |  |  |  |  |
| Technician/Research Assistant | 2.0 | 0.5 | 46.9 | 46.88 |  |
| Nose clip | 1.0 |  | 0.8 | 0.84 | Single use |
| Mouthpiece | 1.0 |  | 2.9 | 2.85 | Single use |
| Bacterial-viral filter -MIP/MEP | 1.0 |  | 2.3 | 2.29 | Single use |
| Plug | 6.0 |  | 100 | 0.12 | 10 Years |
| Two-way non-rebreather valve |  |  | 498.9 | 0.10 | 10 Years |
| Pneumotach HD/Heater |  |  | 1,675.4 | 0.32 | 10 Years |
| Face Tissue | 10.0 |  | 0.03 | 0.26 | Single use |
| Gloves | 6.0 |  | 0.2 | 1.20 | Single use |
| Mask | 3.0 |  | 2.0 | 6.00 | Single use |
| Shield | 2.0 |  | 3.0 | 5.98 | Single use |
| Gown | 1.0 |  | 55.0 | 0.02 | 5 Years |
| CED 1902, 1401, and spike set up | 1.0 |  | 32,818.0 | 6.31 | 10 Years |
| TOTAL |  |  |  | 73.16 |  |
